# Use of diagnostic self-tests on body materials among Internet users in the Netherlands: prevalence and correlates of use

**DOI:** 10.1186/1471-2458-9-100

**Published:** 2009-04-09

**Authors:** Gaby Ronda, Piet Portegijs, Geert-Jan Dinant, Frank Buntinx, Roelf Norg, Trudy van der Weijden

**Affiliations:** 1 Department of General Practice, Faculty of Health, Medicine and Life Sciences, School for Public Health and Primary Care (Caphri), Maastricht University, PO Box 616, 6200 MD Maastricht, The Netherlands

## Abstract

**Background:**

A range of self-tests on body materials has become available to the general public, but the extent of their use has hardly been studied. This study examined how many people use diagnostic self-tests on body materials such as blood or urine, as well as the type of tests that are used, and factors associated with their use.

**Methods:**

Cross-sectional survey. Participants were recruited from an existing Dutch Internet panel of 12,529 persons, and information was collected by means of a structured Internet-based questionnaire. Multiple logistic regression analyses were used to assess correlates of self-test use.

**Results:**

Response to the survey was 63%. Sixteen percent of the respondents said they had ever used at least one self-test, with a mean of 2.1 tests per self-tester. The most frequently reported self-tests were those for diabetes and cholesterol. Self-testers generally reported lower health status and had a higher BMI than non-testers. On the other hand, they were more likely to engage in health-related behaviour such as the use of dietary supplements and homeopathic medicine.

**Conclusion:**

Self-testing proved to be relatively prevalent among Dutch Internet users. We therefore think that it is essential to develop appropriate information for consumers, health care providers and policymakers, about the pros and cons of self-testing and specific self-tests. More test-specific research is needed.

## Background

A range of self-tests on body materials has become available to the general public in the Netherlands in recent years. In an Internet search we found self-tests for over 25 conditions, including cancers, infectious diseases and cardiovascular diseases [1]. These self-tests can be bought over the counter or via the Internet, and require a sample of body material, such as blood, urine, faeces or saliva. We identified four types of self-test: (1) those where the results of the sample are immediately available at home, (2) those where the sample has to be sent to a laboratory with results returned by post or Internet or (3) those where the consumer may have to go to a laboratory to have a sample taken, with results returned by post or Internet. A fourth self-testing option is that of so-called street-corner tests, i.e. tests offered by organizations to consumers in public places like supermarkets. In a street-corner test, a sample is taken by trained personnel, and results are made available immediately. All these tests are offered directly to consumers without the need for a doctor's recommendation. In the true self-test situation at home, the consumer is responsible for all aspects of the tests: execution, interpretation and follow-up behaviour.

Self-testing seems to fit in with current views about consumer autonomy and self-management, and may empower consumers to assume control over their own health care. Self-tests can offer privacy, convenience and reassurance to consumers, as well as earlier diagnosis and treatment. On the other hand, self-tests can cause distress when they yield false-positive results. They can also cause delay of treatment in the case of false-negative results, as well as in the case of true-negative results, when certain symptoms are actually due to another condition [2-6].

Dutch media have reported that the use of self-tests is increasing, but the extent of their use in the Netherlands has hardly been studied. Self-tests are likely to become more easily available and more widely used as the proportion of people having access to the Internet continues to increase [7]. Self-testing, and especially an increase in self-testing, may have important implications for the use of health care services by self-testers and for consumer education about the use and value of self-tests. The first priority, however, is to gain information about the extent of self-test use.

The aim of the present study was therefore to investigate the prevalence of the use of self-tests, as well as the type of tests that are most frequently used, and the association between demographic factors, health-related lifestyle factors, health status and self-test use. Only those self-tests were studied that involve in-vitro tests on body materials, and that are initiated by consumers with the aim of diagnosing a particular disorder or condition. Pregnancy tests were excluded from this survey.

## Methods

### Study population and procedure

Participants were recruited from an existing Internet panel that is managed by Flycatcher, an institute for online research associated with Maastricht University. Anyone who is aged 12 years or over and has an e-mail address can apply to join the panel via the Flycatcher website . Recruitment of panel members takes place through various channels: 'send-to-a-friend' campaigns among existing panel members, newsletters, databases with addresses kept by third parties (after permission), other private panels (after permission), and mouth-to-mouth advertising. If certain subgroups are underrepresented, special campaigns are organized, such as a 'send-to-a-friend' campaign aimed at the specific subgroup. Panel members are invited to participate in a study by being sent an e-mail explaining what respondents are expected to do, what the topic of the questionnaire is, and how much time it will take to fill in the questionnaire. An incentive for participation is offered in the form of a gift voucher that panel members can earn by responding to several questionnaires.

The Flycatcher institute offers the opportunity to draw a sample from the entire panel, which is representative of the Dutch population. To this end, important background variables of panel members (e.g., age, sex, education, postal code, nationality and country of origin) are compared with the latest data from Statistics Netherlands. Compared with the Dutch population, the entire panel is younger, includes more women and is more highly educated [8].

Since the extent of self-test use was unknown, we decided to use the entire panel to ensure that we would discover a reasonable number of self-testers. In September 2006, a short questionnaire in Dutch was thus sent to 12,529 persons aged 12 and over. Self-tests were defined in the introduction to the questionnaire, which specifically mentioned that pregnancy tests and monitoring tests were excluded from the survey. The questionnaire remained online for 8 days. A reminder was sent to non-responders after 5 days. Ethical approval for the study was obtained from the Medical Ethical Commission of Maastricht University/Hospital.

### Questionnaire

A literature review we had performed in preparation to the study revealed a lack of published research on self-tests and the frequency of their use and associated factors, and consequently also on questionnaires about self-testing. We therefore assessed self-test use and possible associated factors with a newly developed short questionnaire. We first conducted an Internet search to identify self-tests that were potentially available to the general public in the Netherlands in 2006, and found self-tests for over 25 conditions, which came in four different types (see Background section). The conditions included in the questionnaire were based on consensus among the research team and external experts. In addition to data about previous and potential future use of self-tests, the questionnaire collected data about demographic and sociodemographic factors, health status and health-related lifestyle factors. The selection of these variables was based on the assumption that they might be associated with self-test use, as previous research had shown that these factors are often related to health-related behaviours in general. Our underlying idea was that it would be especially the 'worried well' who might use self-tests.

The questionnaire was piloted among a small sample (n = 15) of the target population to assess its readability and comprehension, and to check for possible technical errors (online research).

The questionnaire assessed the following variables (see additional file 1: Questionnaire on Self-tests).

1) Whether the person had ever used, had ever considered using, or intended to use a self-test for 25 specified conditions, with room provided to add any tests not listed.

2) Demographic factors: age, sex, weight, height, education, nationality and country of origin.

3) Lifestyle factors: smoking behaviour, physical activity, consumption of fruit and vegetables, dietary fat intake (in terms of types and amounts of fat), alcohol consumption, use of dietary supplements, use of homeopathic medicine and blood donorship.

4) Health status: chronic illness or disability and perceived health.

### Statistical analysis

Basic descriptive statistics were used to describe the use of self-tests, type of self-tests and characteristics of the respondents. Chi-square test statistics were used to assess associations between characteristics of respondents and self-test use. A multiple logistic regression analysis was conducted to identify potential selection bias due to non-response (with response versus non-response as the dependent variable and age, sex and education as independent variables). Further multiple logistic regression analyses were conducted to assess the relationship between self-test use and the various variables. Since we found some significant interactions between sex and the other independent variables, and because there were several sex-specific tests, separate analyses were done for women and men. Differences were considered to be statistically significant if p < 0.05 (two-sided). Analyses were performed with SPSS (Version 13.0).

## Results

### Respondents

Response to the survey was 63% (n = 7919). The mean age of the respondents was 36.7 years (SD = 13.7), with age ranging range from 12 to 94. There were more women (65%) than men (35%) among the respondents. Seventeen percent of the respondents had a low level of education, 39% an intermediate level, and 44% a high level. Non-respondents were found to be younger and less educated than respondents. Almost all respondents had been born in the Netherlands (96%) and had a Dutch passport (98%). More than a quarter (26%) indicated that they had a chronic illness or disability. More than a quarter (27%) perceived their health as moderate (23%) or poor (4%).

### Frequency of self-testing in general and for specific self-tests

Sixty-three percent of the respondents (n = 5019) had ever, i.e. before the present study, heard of diagnostic self-tests. Twenty-eight percent (n = 2220) had ever considered using a self-test and 16% (n = 1263) had used at least one self-test. The mean number of self-tests among self-testers was 2.1, and 9% had used more than four self-tests. The ten most frequently reported self-tests were tests for diabetes, cholesterol, allergies, urinary infection, HIV infection, anaemia, ovulation, Chlamydia, glandular fever and hepatitis. Relevant true home tests included those for diabetes, cholesterol, ovulation, menopause and female fertility, and kidney diseases (Table 1). Three quarters of these true home tests were bought from a chemist, pharmacy or supermarket. The remaining ones were ordered through the Internet, newspapers or magazines (see additional file 1: Questionnaire on Self-tests, question 3b).

**Table 1:** Reported use of a self-test for 25 conditions, frequency of use and frequency of true home testing

Self-tests	number	% of self-testers (n = 1263)	% of total (n = 7919)	number (%^b^) true home tests
- diabetes	488	39	6.2	148 (30)
- cholesterol	431	34	5.4	106 (25)
- allergies	156	12	2.0	21 (14)
- urinary infection	150	12	1.9	31 (21)
- HIV infection	140	11	1.8	9 (6)
- anaemia	136	11	1.7	9 (7)
- ovulation	130	15*	2.5*	119 (92)
- Chlamydia	113	9	1.4	6 (5)
- glandular fever	106	8	1.3	17 (16)
- hepatitis B/C	92	7	1.2	4 (4)
- menopause/female fertility	86	10*	1.7*	65 (76)
- syphilis	67	5	0.8	2 (3)
- vaginal infection/Candida	61	7*	1.2*	8 (13)
- kidney diseases	53	4	0.7	19 (36)
- thyroid diseases	53	4	0.7	1 (2)
- influenza	43	3	0.5	9 (21)
- blood coagulation	42	3	0.5	6 (14)
- fertility male	30	8**	1.1**	6 (20)
- intestinal cancer	28	2	0.4	3 (11)
- prostate cancer	22	6**	0.8**	2 (9)
- test kit for specific diseases^a^	22	2	0.3	3 (16)
- general test kit^a^	20	2	0.3	3 (21)
- hereditary disease(s)	19	2	0.2	1 (7)
- helicobacter pylori	18	1	0.2	3 (17)
- lactose intolerance	14	1	0.2	3 (21)
- gluten intolerance	13	1	0.2	1 (8)
- loss of amniotic fluid	11	1*	0.2*	1 (9)

* % of women

** % of men

^a ^multiple tests per kit

^b ^% of total number of respondents who reported to have used this specific self-test

Seventeen percent of those who had never used a self-test said they would probably or definitely use one in the future and 54% said they would perhaps do so. Seventy-eight percent of these respondents mentioned home tests as their preferred format.

### Differences between self-testers and non-testers

Potential differences in variables between self-testers and non-testers were calculated separately for women and men. Both female and male self-testers, when compared with non-testers, were older (p < .001), had a higher BMI (p < .001), were eating more fruit (p < .01) and less fat (p < .001), were more likely to use dietary supplements (p < .001) and homeopathic medicine (p < .001), were more likely to be blood donors (women: p < .01; men: p < .05), were more likely to report having a chronic disease (p < .001), and perceived to be in poorer health (p < .001). In addition, female self-testers were eating more vegetables (p < .05), whereas male self-testers were less likely to be physically active (p < .05).

### Correlates of self-test use in general

Multiple logistic regression analyses were conducted to assess the relationship between self-test use and the various variables, for women and men (Table 2). Both female and male self-testers, when compared with non-testers, had a higher BMI, were eating less fat (or less saturated fat), were more likely to use dietary supplements and homeopathic medicine, were more likely to report having a chronic disease, and perceived to be in poorer health. In addition, female self-testers had a higher level of education and were more likely to be blood donors, whereas male self-testers were less likely to be physically active.

**Table 2:** Frequency and multivariable odds ratios (OR) with 95% confidence intervals (CI) for correlates of self-test use in general for women and men

	Women^a^			Men^b^		
	
Characteristics	Total N	Self-tester %	Adjusted OR (95% CI)	Total N	Self-tester %	Adjusted OR (95% CI)
	
**Age **(years)						
- ≤ 27	1901	14.3	Reference	736	9.1	Reference
- 28 – 41	1881	18.4	1.12 (0.93–1.35)	822	11.8	1.04 (0.74–1.48)
- ≥ 42	1333	21.2	1.20 (0.97–1.49)	1246	16.1	1.22 (0.86–1.73)
**Educational level**						
- low	940	15.4	Reference	421	12.6	Reference
- intermediate	2124	18.5	1.43 (1.15–1.78)**	979	12.5	1.07 (0.75–1.54)
- high	2051	17.6	1.38 (1.09–1.73)**	1404	13.5	1.19 (0.84–1.68)
**BMI **(weight/height^2^)						
-< 25	3109	15.3	Reference	1411	10.4	Reference
- 25–29.9	1257	20.0	1.28 (1.07–1.54)**	1031	13.3	1.12 (0.86–1.47)
- ≥ 30	721	23.2	1.51 (1.21–1.88)***	359	22.0	1.77 (1.26–2.49)**
**Smoking**						
- no	1382	17.2	Reference	2120	12.8	Reference
- yes	3732	18.5	1.09 (0.92–1.29)	684	13.5	1.02 (0.78–1.34)
**Physical activity **(30 minutes/day)						
- 0 to 4 days a week	2422	16.9	Reference	1462	14.5	Reference
- 5 to 7 days a week	2693	18.2	1.13 (0.97–1.31)	1342	11.3	0.78 (0.62–0.99)*
**Vegetables **(200 grams a day)						
- 0 to 4 days a week	2249	16.2	Reference	1244	12.6	Reference
- 5 to 7 days a week	2866	18.7	1.03 (0.88–1.21)	1560	13.3	1.02 (0.80–1.32)
**Fruits **(2 pieces a day)						
- 0 to 4 days a week	3000	16.3	Reference	1838	11.6	Reference
- 5 to 7 days a week	2115	19.4	1.14 (0.97–1.34)	966	15.5	1.13 (0.88–1.46)
**Fat intake **(in terms of type/amount)						
- 0 to 4 days a week	1930	14.5	Reference	1381	10.6	Reference
- 5 to 7 days a week	3185	19.5	1.22 (1.03–1.44)*	1423	15.3	1.32 (1.03–1.69)*
**Alcohol **(glasses/week)						
- none	2327	17.4	Reference	629	14.5	Reference
- 1 to 14; 1 to 21^c^	2529	17.2	1.05 (0.89–1.23)	1935	12.7	0.93 (0.70–1.22)
-> 14; > 21^c^	259	22.4	1.37 (0.99–1.91)	240	11.3	0.78 (0.48–1.27)
**Dietary supplements**						
- no	2101	13.3	Reference	1554	9.5	Reference
- yes	3014	20.6	1.41 (1.19–1.67)***	1250	17.4	1.75 (1.38-–2.22)***
**Homeopathic medicine**						
- no	3717	15.2	Reference	2369	11.3	Reference
- yes	1398	23.8	1.48 (1.25–1.74)***	435	22.3	1.75 (1.33–2.31)***
**Blood donor**						
- no	4083	16.7	Reference	2101	12.1	Reference
- yes	1032	21.2	1.23 (1.03–1.47)*	703	15.6	1.16 (0.90–1.51)
**Chronic illness or disability**						
- no	3734	15.4	Reference	2121	11.0	Reference
- yes	1381	23.5	1.34 (1.12–1.60)**	683	19.2	1.33 (1.02–1.75)*
**Perceived health**						
- good	3739	15.8	Reference	2073	10.6	Reference
- moderate/poor	1376	22.5	1.25 (1.04–1.49)*	731	19.7	1.53 (1.17–2.00)**

^a ^Women: N total = 5115, self-testers = 899, non-testers = 4216

^b ^Men: N total = 2804, self-testers n = 364, non-testers n = 2440

^c ^14 glasses a week for women, 21 glasses a week for men

* p < .05

** p < .01

*** p < .001

## Discussion

We found that 16% of a sample of Internet users had ever used a self-test, with an average of 2.1 tests per self-tester. Seventeen percent of those who had never used a self-test reported that they probably or definitely would consider using one in the future. The top 10 of most frequently used tests included those for diabetes, cholesterol, allergies, urinary infection, HIV infection, anaemia, ovulation, Chlamydia, glandular fever and hepatitis B/C. Self-testers generally reported lower health status and had a higher BMI than non-testers. On the other hand (or consequently), they were more likely to engage in health-related behaviour such as the use of dietary supplements and homeopathic medicine.

As far as we know, few results of comparable studies on this topic are currently available. A recently published study in the United Kingdom found that almost a third of the respondents had used a self-test, including pregnancy tests and blood pressure monitors. After the exclusion of these two tests, the most frequently reported self-test was that for diabetes [9]. Another study in the United Kingdom, with aims similar to those of our study, is still in progress [2].

As mentioned in the introduction, it is quite possible that consumers with complaints refrain from seeking a doctor's advice in the case of false-negative test results, as well as in the case of true-negative results. We found no studies that had investigated consumers' follow-up behaviour based on the test result. A secondary analysis of a subgroup (n = 684) of self-testers in our study showed that almost one quarter had been confronted with a positive test result, resulting in 75% of them seeking a doctor's advice. Of those reporting a normal test result (72%), 9% reported a follow-up contact with a general practitioner [1]. However, it remains unclear whether there was problematic follow-up behaviour based on the test results.

The present study had several limitations. The generalisability of our Internet sample is clearly an issue. First, our panel is not representative of the Dutch population. Compared with the Dutch population, respondents were younger, more often female, and more highly educated [8]. Next, a non-respondent analysis revealed that non-respondents were younger and less highly educated than respondents. As it can be assumed that the use of self-tests is higher among Internet users than among non-Internet users [10], we assume that the use of self-tests among our Internet sample is higher than among the general Dutch population. Furthermore, some respondents might not have noticed or understood that the survey was limited to diagnostic self-tests, and might also have reported monitoring tests. Although the extent of this bias remains unclear, we expect it to be limited, for two reasons. First, we provided instructions in the questionnaire on the definition of diagnostic self-test (see additional file 1: Questionnaire on Self-tests). Second, a secondary analysis of a subgroup of self-testers in our study revealed that their response to the (open-ended) question about their main reason for self-testing gave little indication of monitoring tests being reported. Another limitation of our study was that it had a cross-sectional design, so no conclusions about causality can be drawn. Finally, our results are based on self-testers in general, and we assessed only a limited number of possible correlates. It is obvious that the characteristics of self-testers and their reasons for testing, as well as the pros and cons of self-tests, are likely to vary with the type of test.

In the Netherlands there is currently a debate about the desirability of self-testing [11]. Whatever the outcome of this debate, self-testing exists and is likely to increase. It is therefore essential to develop appropriate information for consumers, as well as for health care providers and policymakers, about the pros and cons of self-testing and of specific self-tests.

## Conclusion

In conclusion, despite the limitations of the study, self-testing proved to be relatively prevalent in the Netherlands and is a phenomenon that cannot be ignored. On the other hand, self-tests and self-testers cannot be seen as homogeneous groups and more test-specific research is needed. In an ongoing study we are investigating trends in the use of self-tests, psychosocial correlates of their use, consumers' information needs and consumers' follow-up behaviour, in general as well as for specific self-tests.

## Competing interests

The authors declare that they have no competing interests.

## Authors' contributions

GR and TvdW were involved in all aspects of the study and drafted the manuscript. PP was involved in the development of the questionnaire and the data analyses. RN was involved in the development of the questionnaire. GD and FB had a consultative role during all aspects of the study. All of the authors contributed substantially to the production of the manuscript and have read and approved the final version.

## Pre-publication history

The pre-publication history for this paper can be accessed here:



## Supplementary Material

Additional file 1 **Self-tests: September 2006**. The file contains a copy of the questionnaire (translated into English) that was sent to the entire panel of 12,529 persons aged 12 years and over. Click here for file
