# Supplementary material for: Use of diagnostic self-tests on body materials among Internet users in the Netherlands: prevalence and correlates of use
Source: BMC Public Health. 2009 Apr 9;9:100. doi: 10.1186/1471-2458-9-100 (PMC2675528; doi:10.1186/1471-2458-9-100)
Supplement: Additional file 1 — Self-tests: September 2006. The file contains a copy of the questionnaire (translated into English) that was sent to the entire panel of 12,529 persons aged 12 years and over. [file 1471-2458-9-100-S1.doc]

**Self-tests**

**September** 2006

**The questionnaire was originally in Dutch and has been translated into English**

More and more tests are becoming available that allow you to examine your own health. In other words, tests that don’t have to be ordered by a doctor. Not much is known about the current use of such tests in the Netherlands and the need for them. In our survey we try to investigate the use of self-tests en find out what factors may influence their use.

What do we mean by self-tests in this survey?

#### By self-test we mean a test on body samples (such as blood, urine, faeces or saliva) that can be used to detect a disease or the risk of getting a disease, and which you carry out, or have carried out, at your own initiative (so not on the advice of your own doctor).

A blood pressure meter is not a self-test in this sense, as it uses no body materials.

Pregnancy tests are excluded from this survey.

It is important that the decision to have the test was taken by you, and not by your doctor.

There are several ways to use self-tests:

1. buying a self-test for home use from a chemist, pharmacy or supermarket, or ordering it by post, via the Internet or a newspaper or magazine coupon, then applying the test yourself en reading the results;

2. visiting some facility (which may also be a supermarket) at your own initiative, having a test done there and getting the results immediately;

3. visiting a laboratory (e.g. at a hospital) at your own initiative, having a test done there, and getting results sent to you by post;

4. sending in a body sample to a laboratory (at your own initiative), where they do a test and send you the results by post.

#### **Questions about self-tests that can be used to detect a disease or the risk of a disease**

**1. Before this survey, had you ever heard of self-tests? (if yes, multiple answers allowed)**

□ yes, I’d heard of self-tests for home use

□ yes, I’d heard about visiting a facility, having a test done there, and getting the results immediately

□ yes, I’d heard about visiting a laboratory to have a body sample taken, and getting the results sent to me by post

□ yes, I’d heard about sending in a body sample to a laboratory, and getting the test results sent to me by post

○ no (respondent is referred to question 4a)

**2. Have you ever considered using a self-test? (if yes, multiple answers allowed)**

□ yes, a self-test for home use

□ yes, visiting a facility, having a test done there and getting the results immediately

□ yes, visiting a laboratory to have a body sample taken, and getting the results sent to me by post

□ yes, sending in a body sample to a laboratory, and getting the test results sent to me by post

○ no (respondent is referred to question 4a)

**3a. Have you ever done a self-test / had a self-test done? (if yes, multiple answers allowed)**

□ cholesterol

□ diabetes

□ prostate cancer

□ HIV infection

□ hepatitis B or C

□ syphilis

□ chlamydia

□ thyroid diseases

□ blood coagulation

□ helicobacter pylori

□ glandular fever

□ allergies

□ gluten intolerance

□ lactose intolerance

□ anaemia

□ influenza

□ ovulation

□ menopause or female fertility

□ heriditary disease(s)

□ intestinal cancer

□ kidney diseases

□ urinary infection

□ loss of amniotic fluid

□ vaginal infection or candida

□ male fertility

□ general test kit, namely …

□ test kit for specific diseases, namely …

□ other tests, namely …

○ no (continue with question 4a)

**3b. Where did you buy / have this/these self-test(s)**?

|  | bought at chemist’s, pharmacy or supermarket and done at home | ordered through the Internet, newspaper or magazine and done at home | had it done at supermarket, chemist’s, pharmacy or sports centre (e.g. in a screening van) | visited laboratory to have sample taken | sent in body sample to a laboratory | other |
| --- | --- | --- | --- | --- | --- | --- |
| cholesterol | ○ | ○ | ○ | ○ | ○ | ○ |
| diabetes | ○ | ○ | ○ | ○ | ○ | ○ |
| etc. [only answers from question 3a listed in table] | ○ | ○ | ○ | ○ | ○ | ○ |

**4a. Do you intend to use self-tests in the future?**

○ definitely not (respondent is referred to question 5)

○ probably not (respondent is referred to question 5)

○ perhaps

○ probably

○ definitely

**4b. What self-test(s) would you consider? (multiple answers allowed)**

□ cholesterol

□ diabetes

□ prostate cancer

□ HIV infection

□ hepatitis B or C

□ syphilis

□ chlamydia

□ thyroid diseases

□ blood coagulation

□ helicobacter pylori

□ glandular fever

□ allergies

□ gluten intolerance

□ lactose intolerance

□ anaemia

□ influenza

□ ovulation

□ menopause or female fertility

□ heriditary disease(s)

□ intestinal cancer

□ kidney diseases

□ urinary infection

□ loss of amniotic fluid

□ vaginal infection or candida

□ male fertility

□ general test kit, namely …

□ test kit for specific diseases, namely …

□ other tests, namely …

**4c. What type(s) of self-test would you consider? (multiple answers allowed)**

□ a self-test for home use

□ visiting a facility, having a test done there, and getting the results immediately

□ visiting a laboratory to have a body sample taken, and getting the results sent to me by post

□ sending in a body sample to a laboratory, and getting the results sent to me by post

Here are some more questions about your health status, lifestyle and personal details.

#### **Health status**

#### **5. How would you rate your own health?**

○ very poor

○ poor

○ reasonable

○ good

○ very good

**6. Do you have any chronic disease, impairment or disability?**

○ yes: …

○ no

#### **Lifestyle factors**

**7. Have you smoked one or more cigarettes (including roll-your-owns), cigars of pipes in the last 7 days?**

○ yes

○ no

**8. On how many days a week are you physically active (e.g. walking, cycling, sports, gardening, or physically demanding household chores like mopping, vacuuming and cleaning windows) for at least 30 minutes a day?**

… days a week [respondents can enter a maximum of 1 digit between 0 and 7]

**9. On how many days a week do you eat at least 200 g (4 serving spoons) of vegetables?**

… days a week [respondents can enter a maximum of 1 digit between 0 and 7]

**10. On how many days a week do you eat at least 2 pieces of fruit?**

… days a week [respondents can enter a maximum of 1 digit between 0 and 7]

**11. On how many days a week do you take the type and quantities of fat in various products into account when deciding what to eat?**

… days a week [respondents can enter a maximum of 1 digit between 0 and 7]

**12. On how many days a week do you usually drink beer, wine or other alcoholic beverages?**

… days a week [respondents can enter a maximum of 1 digit between 0 and 7]

**13. How many glasses do you usually drink on such a day? If you don’t drink any alcohol, please fill in 0.**

… glasses a day [respondents can enter only figures]

**14. Do you use any nutritional supplements (extra vitamins and/or minerals and suchlike)?**

○ yes, throughout the year

○ yes, in certain periods (e.g. in winter)

○ yes, but only when my disease resistance is low / I don’t feel well

○ yes, other: …

○ no

**15. Do you use any homeopathic medicines to increase your disease resistance or improve your health?**

○ yes, throughout the year

○ yes, in certain periods (e.g. in winter)

○ yes, but only when my disease resistance is low / I don’t feel well

○ yes, other: …

○ no

**16. Are you, or have you ever been, a blood donor?**

○ yes, I’m currently a blood donor

○ yes, I’ve donated blood in the past

○ no, I’ve never donated blood

# Demographic factors

**17. When were you born?**

….

**18. Are you a man or a woman?**

○ man

○ women

**19. What is your height (in centimetres)?**

… cm [respondents can enter a maximum of 3 digits between 100 and 250]

**20. What is your weight (in kilogrammes)?**

… kg [respondents can enter a maximum of 3 digits between 0 and 999]

**21. What is your level of education (that is, the highest type of school from which you have received a diploma (except primary school))**

○ Low level: primary school or basic vocational school

○ Medium level: secondary vocational school or high school degree

○ High level: higher vocational school or university degree

**22. In what country were you born?**

○ The Netherlands

○ Belgium

○ Germany

○ Morocco

○ Surinam

○ Turkey

○ other: …

**21. What nationality do you have?**

○ Dutch

○ Belgian

○ German

○ Moroccan

○ Surinam

○ Turkish

○ other: …
